# Characterization of ATP10B R303W as a Novel Pathogenic Mutation in Parkinson's Disease

**DOI:** 10.1002/alz70855_097660

**Published:** 2025-12-23

**Authors:** Danyeong Kim, Da‐Eun Jeong, Yunseo Gong, Hongjae Ahn, Min Ju Kang, Seong Soo A An

**Affiliations:** ^1^ Veterans Health Service Medical Center, Seoul, Korea, Republic of (South); ^2^ Department of Bionano Technology, Gachon University, Seongnam, Korea, Republic of (South)

## Abstract

**Background:**

ATP10B encoded a phospholipid flippase, essential for maintaining cellular homeostasis, which was implicated in the pathogenesis of Parkinson's disease (PD). Previous studies suggested an association between ATP10B dysfunction and PD, still the relationship between ATP10B and alpha‐synuclein remained unclear. In this study, a novel ATP10B R303W mutation was identified in a patient with familial PD. While ATP10B was linked to PD, this would the first study to explore its role in alpha‐synuclein metabolism, a critical hallmark of PD pathology. By establishing cellular model of expressing ATP10B R303W, its effects on mitochondrial and lysosomal functions and alpha‐synuclein were investigated.

**Method:**

CRISPR/Cas9 technology was used to transfect HEK293 cells with ATP10B R303W and SNCA A53T, as a positive control. Successful transfections were confirmed by Sanger sequencing. ATP10B and LC3 gene expressions were analyzed by RT‐qPCR. Alpha‐synuclein levels in the supernatant, cytosol, and membrane fractions were measured. Mitochondrial dysfunction was assessed using TMRE and MitoTracker, and lysosomal dysfunction was evaluated by monitoring pH changes, and enzyme activity. Alpha‐synuclein phagocytosis was analyzed by confocal microscopy.

**Result:**

ATP10B R303W and SNCA A53T mutations were successfully generated. Compared to HEK293 cells (negative control), ATP10B and LC3 gene expressions were reduced in ATP10B R303W and SNCA A53T cells. Alpha‐synuclein levels were elevated in cytosolic and membrane fractions of ATP10B R303W and SNCA A53T cells, but decreased in the supernatants of ATP10B R303W cells. Both mutations exhibited reduced mitochondrial membrane potentials. ATP10B R303W cells also showed the increased lysosomal pH, and reduced enzyme activities. Confocal microscopy confirmed the diminished alpha‐synuclein phagocytosis in ATP10B R303W cells.

**Conclusion:**

This study established ATP10B R303W as a novel pathogenic associated mutation with PD. We demonstrated that ATP10B R303W disrupted mitochondrial and lysosomal functions, increasing cytosolic and membrane‐bound alpha‐synuclein while reducing its secretion and phagocytosis. These findings directly linked the ATP10B dysfunctions to alpha‐synuclein dynamics, contributing to PD pathology. The familial nature of this mutation underscored its relevance in hereditary PD. Future studies should target ATP10B to restore cellular homeostasis and mitigate alpha‐synuclein aggregation.

